# Point-of-Care Virtual Surgical Planning and 3D Printing in Oral and Cranio-Maxillofacial Surgery: A Narrative Review

**DOI:** 10.3390/jcm11226625

**Published:** 2022-11-08

**Authors:** Daniel Ostaș, Oana Almășan, Robert R. Ileșan, Vlad Andrei, Florian M. Thieringer, Mihaela Hedeșiu, Horațiu Rotar

**Affiliations:** 1 Department of Oral and Cranio-Maxillofacial Surgery, “Iuliu Hațieganu” University of Medicine and Pharmacy, 33 Moților Street, 400001 Cluj-Napoca, Romania; 2 Department of Prosthetic Dentistry and Dental Materials, “Iuliu Hațieganu” University of Medicine and Pharmacy, 32 Clinicilor Street, 400006 Cluj-Napoca, Romania; 3 Department of Oral and Cranio-Maxillofacial Surgery, University Hospital Basel, 21 Spitalstrasse, 4031 Basel, Switzerland; 4 Medical Additive Manufacturing Research Group (Swiss MAM), Department of Biomedical Engineering, University of Basel, 16 Gewerbestrasse, 4123 Allschwil, Switzerland; 5 Department of Oral Rehabilitation, Faculty of Dentistry, “Iuliu Hațieganu” University of Medicine and Pharmacy, 15 Victor Babes Street, 400012 Cluj-Napoca, Romania; 6 Department of Maxillofacial Surgery and Implantology, “Iuliu Hațieganu” University of Medicine and Pharmacy, 37 Cardinal Iuliu Hossu, 400029 Cluj-Napoca, Romania

**Keywords:** 3D printing, point-of-care, virtual surgical planning, additive manufacturing, maxillofacial surgery, cranial surgery, in-house 3D printing, hospital-based printing

## Abstract

This paper provides an overview on the use of virtual surgical planning (VSP) and point-of-care 3D printing (POC 3DP) in oral and cranio-maxillofacial (CMF) surgery based on a literature review. The authors searched PubMed, Web of Science, and Embase to find papers published between January 2015 and February 2022 in English, which describe human applications of POC 3DP in CMF surgery, resulting in 63 articles being included. The main review findings were as follows: most used clinical applications were anatomical models and cutting guides; production took place in-house or as “in-house—outsourced” workflows; the surgeon alone was involved in POC 3DP in 36 papers; the use of free versus paid planning software was balanced (50.72% vs. 49.27%); average planning time was 4.44 h; overall operating time decreased and outcomes were favorable, though evidence-based studies were limited; and finally, the heterogenous cost reports made a comprehensive financial analysis difficult. Overall, the development of in-house 3D printed devices supports CMF surgery, and encouraging results indicate that the technology has matured considerably.

## 1. Introduction

The complex anatomy and functionality of the craniofacial structures, together with the pursuit of the best clinical outcome, demand state-of-the-art, patient-specific treatments. Though three-dimensional printing (3DP) has been around since 1986, the technology became highly visible once medical researchers began exploring 3DP and its role in personalized medicine [1]. Companies, research facilities, hospital-based 3DP laboratories, or the associations of the previously mentioned entities produce patient-specific surgical devices for oral and cranio-maxillofacial (CMF) surgery [2,3,4]. Externalized virtual surgical planning (VSP) and 3DP can be considered expensive, with a significant financial impact on the healthcare system [5]. They are also time-consuming, causing problematic delays for urgent cases [6]. A universally accepted definition of point-of-care 3DP (POC 3DP) is difficult to provide; however, literature defines it as the just-in-time creation of 3D printed anatomic models, surgical instruments, or other medical devices based on the patient’s imaging data, either at the place of patient care or in a facility owned by the health care provider [7]. Special efforts have been made so that surgeons can directly manufacture patient-specific devices at the POC in order to cope with urgent medical demands and reduce the economic impact that these technologies have on the healthcare system [8,9].

In medicine, analytic investigations on 3DP are conducted on a wide spectrum of surgical specialties—orthopedics, spinal surgery, maxillofacial surgery, neurosurgery, and cardiac surgery—which are, generally, analyzed together [10]. Despite CMF surgery’s influential role in the development and use of additive technologies, few studies have focused strictly on the analysis of in-house VSP and 3D printing of this specialty [2,10,11,12,13,14,15,16].

This paper aims at providing an overview on the usage of virtual surgical planning and 3D printing at the point-of-care in CMF surgery based on a review of articles from three major literature databases. We focused our investigation on the following parameters: clinical applications, infrastructure, the time necessary for planning/printing, operating time, cost, and outcomes.

## 2. Materials and Methods

### 2.1. Information Sources

We structured a search in the electronic databases of PubMed, Web of Science, and Embase on articles published between January 2015 and February 2022, and performed the final electronic search on all databases in March 2022.

### 2.2. Search Strategy

The following terms were searched: “3D printing”, “three-dimensional printing”, “additive manufacturing”, “maxillofacial surgery”, “cranial surgery”, “in-house”, and “hospital printed”, in combination with the Boolean operator “AND”. To find all possible combinations of papers, we performed twelve separate searches. For the complete search strategy for PubMed database, see Supplementary Table S1. A manual search of the identified articles was also conducted.

### 2.3. Eligibility Criteria

The selection criteria included publications that described the human application of virtual surgical planning and 3D printing, were released between January 2015 and February 2022, were available in full text, and were written in the English language. We excluded papers that had no hospital-based potential, studies on dental applications and bioprinting, reviews, and duplicates. Manual title and abstract screening were done immediately after electronic filters were applied, thereby eliminating duplicates. Any further missed duplicates were removed when papers were introduced in Mendeley (Mendeley Software, London, UK), a bibliographic software used to acquire and arrange all references. Furthermore, we retained titles containing “low-cost”, “entry-level”, “in-office”, “office-based”, “surgeon driven”, “self-made”, and “open source” so as to not overlook potential uses in the hospital environment. The selected eligible papers went through a full-text overview, and we analyzed the ones selected in detail using an Excel evidence table to report relevant study characteristics.

### 2.4. Data Collection Process

Data were extracted using a standardized form, which included the following information: (1) authors’ names and publication year, (2) clinical application, (3) accommodation of infrastructure, (4) human resources involved, (5) software, (6) hardware and materials, (7) planning time, (8) production (3DP time), (9) operating room time, (10) cost, and (11) outcome (Supplementary Table S2).

## 3. Results

### 3.1. Selection of Sources of Evidence

**Figure 1:**
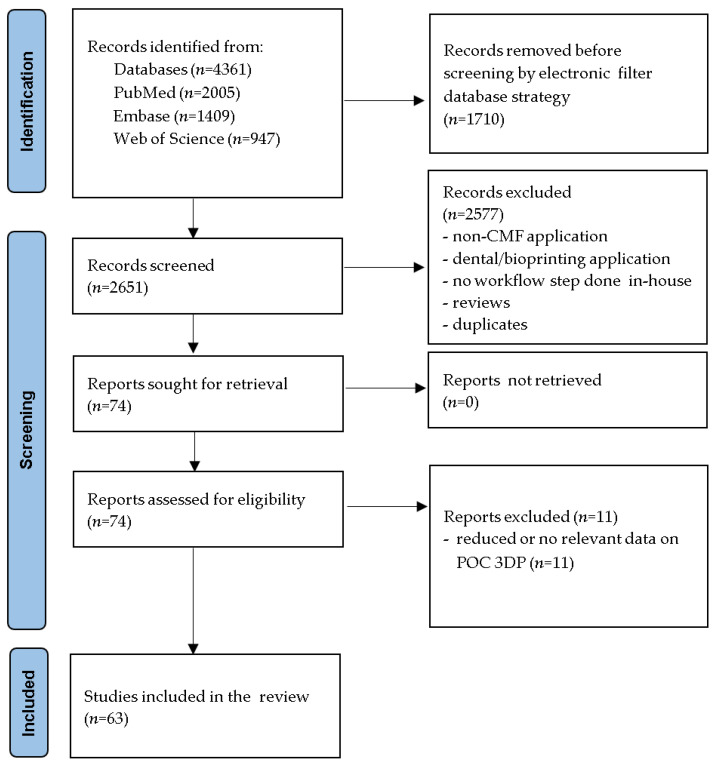
Schematic representation of the strategy for the selection of final articles.

The database search, using the keywords previously mentioned without any filters, resulted in 4361 papers. After applying electronic database filters (inclusion criteria), we retained 2651 papers. The manual screening of titles and abstracts resulted in the exclusion of 2577 articles, leading to 74 eligible articles. Eleven papers were excluded from the analysis due to reduced or no relevant data referring to POC 3DP. The included studies were case reports, case series, and technical notes with a retrospective review of relevant data. No authors clearly described a prospective study design in the selected papers. Finally, the review included a total of 63 articles. The search strategy is evidenced in Figure 1.

### 3.2. Clinical Applications

Anatomical models were the most common patient-specific devices planned and produced at the point-of-care. These were used for preoperative planning in cases of complex anatomy, such as arterio-venous malformations, pre-bending osteosynthesis gear (metal plates, meshes), or pre-forming grafts [6,9,17,18,19,20,21,22,23,24,25,26,27,28,29,30,31,32,33,34,35,36]. The utility of 3D models extended to patient information and consent, the education of medical staff, quality control, or forensics [37,38].

Models were followed by cutting/positioning guides that address mandibular and maxillary reconstructions with the help of vascularized fibula, iliac crest, or scapular grafts [3,4,19,39,40,41,42,43,44,45,46,47,48,49,50,51,52,53,54,55,56].

Cranioplasty plates used for cranial reconstructions were commonly produced in the hospital of treatment either by directly printing molds or by printing the cranial plate template based on which a silicon mold is obtained. Polymethylmethacrylate (PMMA) was the material of choice used for the fabrication of cranial implants through this procedure [57,58,59,60,61,62]. Molds were also used for stenting meshes used for orbital reconstructions [53,63,64,65].

**Figure 2:**
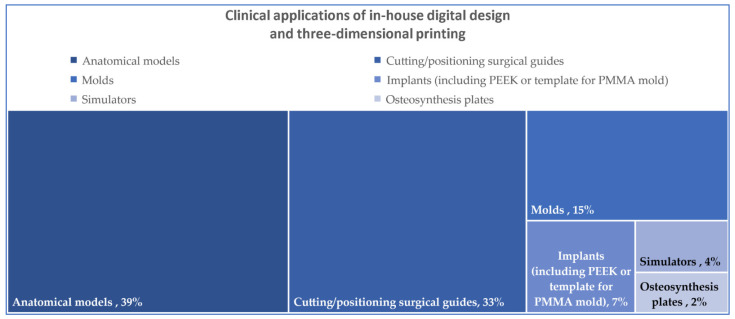
In-house 3DP of clinical applications across the studies and their percentual distribution.

One way to address the in-house production of patient-specific medical devices was reported by Yang et al. (2020), who designed a prototype of the patient-specific osteosynthesis plate that was sent to engineers who optimized the final product [4]. Implantable devices with a full in-house workflow are not common but efforts are being made towards their development. Philipp Honigmann et al. (2018) reported the experimental production of implantable devices (osteosynthesis plate, cranioplasty plate, midface-zygomatic bone) with the help of fused filament fabrication technology (FFF)—all with potential in-house production [66]. A percentual distribution of the in-house clinical applications mentioned across all articles can be consulted in Figure 2.

### 3.3. Infrastructure

#### 3.3.1. Housing of Virtual Planning and 3D Printing Infrastructure

The analysis of the data showed inhomogeneous reporting regarding the housing of planning and 3D printing infrastructure. Some authors mentioned that the process of production took place in the treatment facility (hospital 3DP laboratories, radiology-based 3D printing facilities, information technology departments) or straightforward as “in-house” [4,6,9,17,18,20,23,25,27,29,30,31,32,33,34,35,36,37,38,40,43,44,45,46,47,48,49,50,51,52,53,54,55,56,59,60,61,62,64,65,67,68,69,70,71]. Some authors did not clearly state the accommodation of infrastructure but rather evoked the in-house concept [19,21,22,39,41,57,72,73,74]. Others reported planning carried out in the institution of treatment, while printing was outsourced [4,26,28,42,63]. In one case, virtual surgical planning was undertaken with a commercial provider via videoconferencing, as is usual for other elective CMF cases. However, instead of being printed by the VSP provider, the resulting stereolithography file (STL) was downloaded and printed in-house [36]. Finally, papers also report work carried out in the laboratory/for research purposes, validating the experimental work for potential in-house use [24,58,66,75].

#### 3.3.2. Software

The scope of the present section is to give an overview on the software solutions used for the POC development of medical devices in CMF surgery. We use brand names that are/can be protected but are not marked with ®. Software solutions, according to the purpose of use, can be classified as segmentation software: e.g., MIMICS Innovation Suite (Materialise Inc., Leuven, Belgium), 3D Slicer [76], and In Vesalius (CTI Renato Archer, Campinas, Brazil); planning software: e.g., 3-matic (Materialise Inc., Leuven, Belgium), Blender (Blender Foundation; Amsterdam, Netherlands), and MeshMixer (Autodesk Inc, California, USA); or software solutions that can do both, such as the powerful CMF ProPlan (Materialise Inc., Leuven, Belgium).

**Figure 3:**
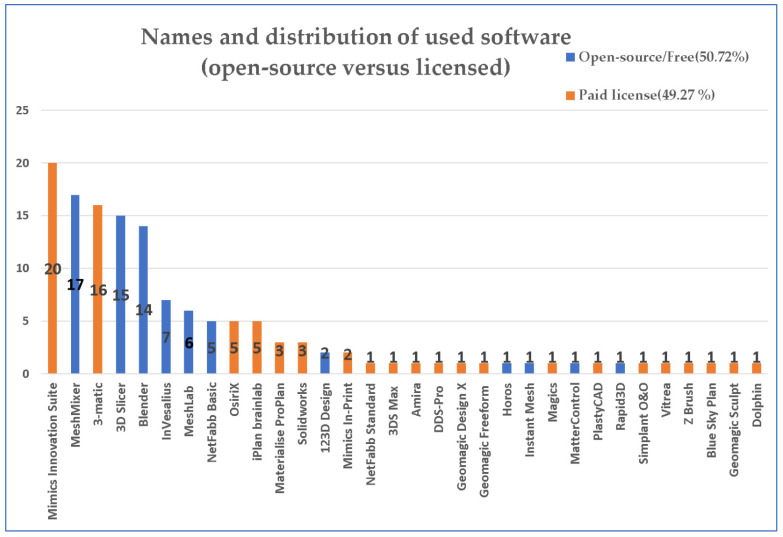
Graphical representation of the used software, number of mentions across the reviewed articles, and percentual usage distribution of free versus paid software.

Some software solutions are easily accessible because they are free (free license or open-source), which makes them useful for point-of-care facilities with a small budget, while others are available only with a paid license. In this review, we will not address the issues surrounding the use of software with or without medical certification, as it is a regulatory issue. Figure 3 depicts an overview of the software solutions utilized, including the type of software (open source/free, paid license), the number of quotes for each software, and the percentual distribution of free and paid software use.

While 21 paid license software and only 11 free software were used, a closer look at the number of times each software was mentioned indicates a balanced ratio between free and paid versions (50.72% free vs. 49.27% paid). For three software (Ayra, Ikeria SARL, Sevilla, Spain; Volume Extractor 3.0, i-Plants systems, Iwate, Japan; and POLYGONALmeister Ver. 4; UEL Corp., Tokyo, Japan) data on license type were undisclosed/unavailable online [31,40].

#### 3.3.3. The 3D Printers and Materials Used at Point-of-Care

Printers that use Fused Deposition Modeling (FDM)/Fused Filament Fabrication (FFF) technology are most used at the point-of-care, likely due to their low price tag. For models, FDM/FFF techniques use PLA or ABS, while PEEK becomes a valuable option for implantable devices [3,9,17,20,21,22,23,24,26,28,29,30,31,32,35,36,37,38,40,44,45,46,47,48,49,50,51,53,57,58,59,63,64,66,71,72,73,74,75]. Stereolithography, Polyjet/MultiJet/MaterialJet, and Digital light processing use resins/photopolymers [3,6,18,19,24,33,34,35,37,39,40,41,51,52,54,55,56,59,60,61,62,65,67,68,69,70]. ColorJet(CJP)/Binder jetting (BJ) printing involves two major components—a core (powder) and a liquid binder [25,55]. These technologies are recognized for their high accuracy, biocompatibility, and sterilization tolerance. Selective laser sintering/melting (SLS/SLM) use powders (polyamide 12 or titanium) to create the final products [4,28,42].

**Figure 4:**
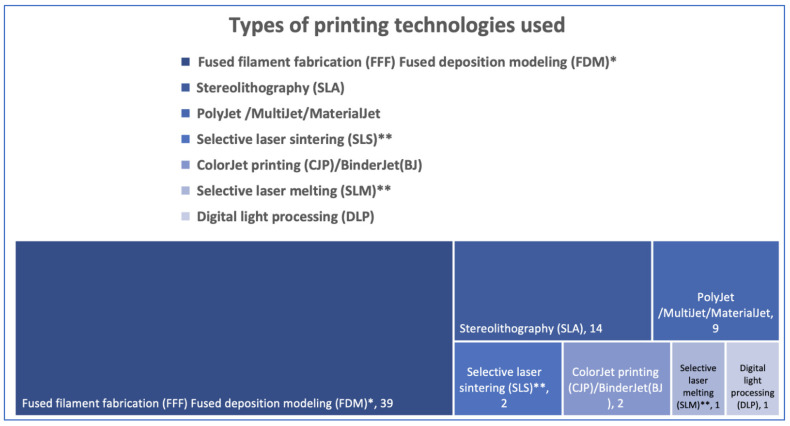
Printing technologies used across the studies (”*”—the same technology; ”**”—described as being part of an in-house process but not yet widely accessible for hospital use).

Figure 4 provides information on 3D printing techniques and frequency of use. Some printing techniques are only mentioned as being part of an in-house process (design carried out in-house with outsourced printing), as they are not yet widely accessible for hospital use (marked with ”**”) [4,28].

### 3.4. Human Resources Involvement

The human resources involved in the process of 3D printing at the point-of-care in a CMF surgery department refer to: the surgeon alone (thirty-six papers), surgeon and radiologist (five papers), surgeon and information technology specialist/bioengineer (six papers), radiologist and technician (two papers), radiologist alone (one paper), and technician alone (one paper). The rest of the papers had no reference to the human resources involved. No reference was encountered concerning who performs administrative tasks, such as the acquisitions of computers, software, consumable materials, or maintenance. Clear details regarding who carries out the printing and post-processing work are also overlooked in the reviewed articles.

### 3.5. Time Management for in-House 3DP Products

#### 3.5.1. Planning Time

Planning time was mentioned in 31 of the 63 evaluated papers. Reports were made in minutes, hours, or days, either as intervals or as an average. Where time intervals were given, the average was calculated. All reports were transformed into hours. The average planning time reported in the 31 papers was 4.44 h, covering segmentation and actual virtual planning. One of the shortest reported planning times was 0.25 h, Evins et al. (2018) needed an average of 14.6 min of virtual planning (from CT data import to printing initiation) for FDM-produced craniofacial prosthesis and molds [58]. The longest average planning time reported was 30 h, due to the production of customized surgical mandibular/fibula osteotomy guides [40]. The hours of planning can span over a few days, as oftentimes surgeons perform this work in their spare time. The rest of the papers did not mention the time spent on planning or were limited to mentioning that the fabrication process took only a few hours, without clear numbers to support their report [28,44]. Other authors focused on reporting the entire production time without making separate time reports on virtual planning, 3D printing, and post-processing [31,35,49,55,60].

Planning time depended on the complexity of the intervention or the necessary learning curve to become accustomed to the software capabilities. Zavattero et al. (2020) needed 6 months to become accustomed to the software [39,48]. Planning time was influenced by the type of software used. Professional software requires less time, while nonprofessional software planning took almost double the time, due to the learning curve and user-friendliness of professional software [24].

#### 3.5.2. Three-Dimensional Printing Time

Actual printing times can range from a few hours to multiple days, as it depends on the printing technique, size, complexity, and number of printed parts [37]. Otherwise, the printing process is automated and does not necessarily require human supervision [26].

Because of multiple printing techniques and applications, as well as the inhomogeneous way authors reported printing time, the data were summarized based on the most common techniques and applications used at the point-of-care. Reports were made in minutes and hours, as time intervals or as averages; where time intervals were given, the average was calculated in hours.

Using FDM/FFF technique, mandibular/maxillary models and cutting guides taken altogether needed an average printing time of 7.8 h [3,9,20,21,23,29,45,46,47,48,49,51,53,75]. Molds/cranial plates took an average printing time of 3–4 h [57,58,72], while an orbital model claimed 24 h (considering printer booting, setting the machine, printing, removing support, and cleaning the piece) [24]. Other applications, such as in-house-made complex head models for preoperative patient education and consultation, surgical planning, and resident training took longer printing times, around 48 h [38]. To obtain a general view of the FDM printing time, Bergeron et al., reported a mean printing time of 7.9 h for clinical applications, such as anatomical models of the cranium, mandible, and orbit, with the printing phase time per model ranging from 2 h 36 min to 26 h 54 min [36].

With the help of SLA technology, authors reported printing cranial plate molds in an average printing time of 6.8 h, with times ranging from 3–5 h for the template ring, 5–8 h for the template mold in the case of the “springform” technique cranioplasty, and up to 10 h for the classical type of cranial plate mold [59,62]. A temporal bone model used as a simulator was printed in 7 h [67], orbital models and molds for orbital implant pre-bending were printed in 11.5 h [65], while mandibular and fibula cutting/positioning guides were printed in an average of 2.52 h [3,51,56].

Few of the remaining printing techniques had reports on printing time but to disclose some of them, we will mention three examples: models of arteriovenous malformations were printed in an average of 9 h (6–12 h) by PolyJet technique, an orbital floor model was printed with the help of MultiJet Printing in 18 h, and a mandibular model was printed with ColorJet Printing in 4.5 h (270 min) and used for reconstruction plate pre-bending [18,24,25].

The shortest reported printing time referred to a mandibular model that was printed using DLP technology in 1 h, with 30 min of post-processing. However, the authors mentioned that this was a prototype printer unavailable to most clinicians and with a substantial price. They suggested that printing the same part with SLA technology would take around 5 to 7 h [6]

Post-processing can be manual or semi-automatic, as it involves—depending on technique—the removal of the support material, sandblasting, light curing, washing, and sterilization. Post-processing is an important part of the production chain but few authors mentioned the necessary amount of time for this process with reports varying from 30 min to 2 h [6,20,46,51,52,67].

#### 3.5.3. Operating Time

In the evaluated papers, the assessment of surgical time regarding procedures that involved point-of-care 3D planning/printing is heterogenic but most state time reduction. We can split the papers into three major categories.

The first and most relevant category refers to papers that reported reduced OR time, backed up by numbers and statistics based on comparisons made between the intervention group (on which the point-of-care 3D printing application was used) and the conventional group [6,18,30,31,35,42]. To exemplify, Weinstock et al. (2015) reported that the surgical time (from initial incision to closure) was 12% faster in the two cases of arterio-venous malformations that used 3D models (on average, approximately 30 min faster with 3D models; non-model cases 285 and 288 min, 3D model cases 254 and 257 min, respectively) [18]. Ganry et al. (2017), using fibula cutting guides planned in-house and printed outsourced, reported that the surgical procedure time was reduced by 1.5 h on average [42]. Marschall et al. (2019) printed reduced mandible models of trauma patients for plate pre-bending and claimed that OR treatment time was 1.5 h versus an approximate time of 2.25 h for traditional open reduction and internal fixation (ORIF) [6]. Using 3D printed anatomical models of the mirrored orbit for the pre-bending of orbital meshes, Sigron et al. (2021) calculated that the mean duration of the surgery was significantly reduced by 35.9 min in the intervention group (58.9 (SD: 20.1) min) compared to the conventional group (94.8 (SD: 33.0) min, *p*-value = 0.003).

A second category of papers reported actual operating room time but without any other calculations to prove operating room time economy (though in some of the papers’ literature data on operating time were taken as reference for comparison) [17,21,25,34,40,41,46,47,56,57,59,60,61,62,69].

The third category of papers discussed reduced operating room time. However, the statement was not backed up by numbers and statistics from the authors’ experience but rather by the literature references, personal suppositions, or expectations [19,20,22,23,26,27,29,32,39,44,48,49]. The rest of the authors did not mention/address the subject of operating room time, or the operating time did not apply to their study.

### 3.6. Costs

The visible published costs for the in-house approach might seem low when authors report just the price of the material used to print a medical device or when some of the costs are omitted, but most of the time the actual costs are higher when all expenses are taken into account [47]. The main costs one should address are rent for housing the infrastructure, human resource expenses (training and surgeon’s time), computer, software license (acquisition and renewal), 3D printer’s price, and running costs (printing material, accessories, and maintenance).

To aid readers in search of pure informative costs, we will provide the reported costs of key elements in the point-of-care production of surgical devices, neglecting the cost to house the infrastructure and price of computers as they were not reported.

Concerning human resources, costs are region specific: Goetze et al. (2017) reported personnel costs of 367 EUR for printing one cutting guide, Legocki et al. (2017) mentioned a 45 USD/hour rate for an information technology employee, while Spaas and Lenssen (2019) based their calculations on the labor cost of a junior surgeon in Belgium at around 15.24 EUR/hour [9,20,39]. Training of personnel can cost 225 USD for a 3 h session in 3D printer operation and can reach 3000 EUR for a 2-day professional training session on how to use a segmentation/3D planning software [17,51].

If one does not use a free software solution, a license price was reported to vary from 300 USD for a lifelong software license (DDS-Pro) [72] to yearly renewable licenses that vary from 699 USD (Osirix) [20] to 12,000 EUR/year (MIMICS) [24]. In another paper, the most commonly used software pack, MIMICS, had a detailed quotation that reached 21,000 USD/year for the three-module configuration (base module, performing segmentation, and 3D reconstruction, costing 5833 USD; a design module, which provides design tools to create devices, 8726 USD, an analysis module costing 6388 USD) [60].

Printer acquisition prices depend on technology, with FDM printers ranging from 600 USD to 5000 USD and above [20,21,23,28,29,36,40,45,47,48,49,51,57,59,73]; only two of the papers that reported prices for FDM printers also reported cost for maintenance (200 USD/year)/printer protection plan (350 USD/year) [17,24]. Stereolithography printers can be purchased at prices that range from 3500 USD to approximately 5000 USD, with all accessories included (UV light, washing machine) [3,51,59,67]. Printers over the prices of 50,000 USD, such as the 3D System ProJet 3510 or the 300,000 USD EOSINT P385 are beyond the budget of most hospitals and can be found either in research laboratories (housed within a hospital) or in outsourced facilities [24,28].

Printing materials are represented by: (1) filaments (Polylactic acid (PLA) with a cost varying from 11.90 to 60 USD/kg [20,21,24,29,51,72,73], while Acrylonitrile butadiene styrene (ABS) is reported to cost around 43 USD/kg [23]; (2) photopolymers (have multiple prices reported: 175 USD/kg, 200 USD/cartridge, 280 EUR/1L, and even 570 USD/2 kg, depending on the indication/properties [6,24,51,67]); and (3) powders (polyamide 12 or titanium), which had no reported costs [4,28,42]. We consider these prices to only be informative, as companies adapt their prices to consumers in accordance to buying power or based on individual deals.

Unfortunately, the heterogeneity of the reported data prevented an in-depth analysis with a true comprehensive cost analysis. By far, the article that most efficiently reported their cost data analysis was published by Abo Sharkh and Makhoul (2019). We consider their example a model of good practice [3].

### 3.7. Outcome of Point-of-Care Virtual Planning and 3D Printing

Parameters related to outcome differed from author to author, and throughout the literature, we did not find a standardized procedure for reporting outcomes. A clear, outcome-based classification of the papers was difficult to create due to the heterogenous way outcomes were reported. However, guided by the evidence-based principles, two categories could be individualized: outcomes backed up by numbers and statistics and outcomes that were not. On the side of outcomes backed up by numbers, the following parameters were highlighted among reported data: accuracy, reduction of operating room time, cost-effectiveness, and blood loss.

The accuracy of the clinical result is of utmost interest, but only 20 out of 63 papers sustained their findings with objective numbers and statistics. In-house-produced fibula and mandibular/maxillary cutting guides were reported as accurate by assessing the reproduction of the planned results in eight papers. All reconstruction procedures were considered successful, with a good match between the digitally planned and the final result of the surgery [4,9,40,42,44,45,51,56].

Concerning the orbit, accuracy evaluation focused on assessing pre- and post-surgical orbital volume, implant fit at the fracture site, and ophthalmic examinations made before surgery and post-operatively [30,32,34,35,53,64].

Chamo et al. (2020) studied in-house cranioplasty implant templates used to create molds, with results suggesting that deviations for the test groups did not exceed 1 mm, which is an acceptable accuracy for clinical routine in craniofacial reconstruction [74]. Tel et al. (2020) showed in numbers the accuracy of cranial reconstructions using cranioplasty plates obtained with the help of 3D printed molds [60]. Sharma et al. (2021) went to the next level and proved that point-of-care FFF 3D-printed PEEK cranial PSIs had high dimensional accuracy and repeatability [71].

Hatz et al. (2019) conducted a study comparing mandibular models printed with entry-level printers accessible in hospital facilities with models printed by industrial printers and found that the accuracy of in-house printed models can serve the surgical management of maxillofacial pathology [28]. Legocki et al. (2017) found similar accuracy results but on a smaller group of neonatal, pediatric, and adult-sized mandibular models [20]. Naros et al. (2018) went further, demonstrating that mandibular models used to pre-bend titanium reconstruction plates accurately reconstruct the symmetry and continuity of resected mandibles [25].

Though operating room time economy was already discussed, we would like to stress again that although multiple studies evoked a reduced surgical time, only six supported their statements with findings and numbers based on comparisons between the group on which the point-of-care 3D printing application was used and the conventional group [6,18,30,31,35,42].

Although cost-efficiency was suggested as a positive outcome by a great number of articles included in the standard analysis, only 10 papers stated cost-efficiency being backed up by numbers [3,9,17,20,28,30,39,47,60,62]. Even so, every cited group of authors has its own standard of analysis (we focused here on papers in which a form of comparison was carried out between investment/expenses and the costs cited in the literature or provided by industry). Therefore, making an in-depth evaluation of cost-efficiency as an outcome at the international level has not been feasible.

Narita et al. (2020) assessed blood loss when using 3D models in orthognathic surgery, reporting a mean amount of bleeding (grams) of 252.2 ± 97.7 g (with 3D models) vs. 331.2 ± 85.9 (without 3D models) [31].

While the rest of the papers reported a good outcome; unfortunately, they were not backed up by numbers or statistics. Besides the parameters priorly mentioned, a good outcome also concerned parameters such as safety of use, efficiency, precision, facial symmetry, or low rate of perioperative complications.

## 4. Discussion

**Figure 5:**
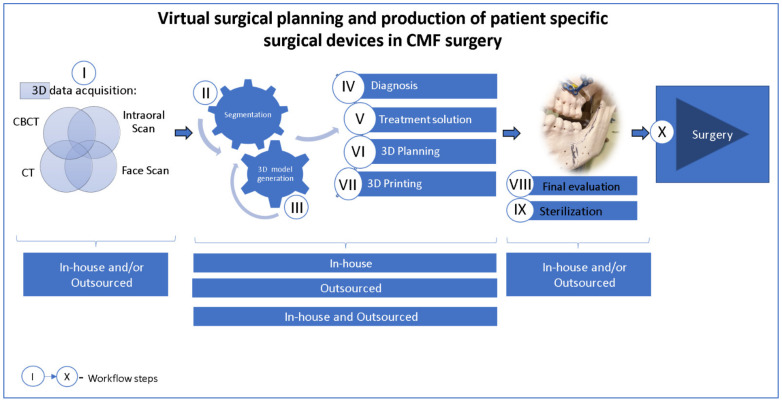
Manufacturing workflow for patient-specific surgical devices in CMF surgery.

In CMF surgery, many organizations (businesses, research centers, hospital 3DP laboratories) work together to accomplish virtual surgical planning and the manufacturing of patient-specific surgical equipment, typically following a process such as the one shown in Figure 5.

Most analytic studies/reviews address virtual surgical planning and additive manufacturing focused on the application of 3D printing in a variety of medical fields. Concerning CMF surgery, in most of the papers, in-house manufacturing was investigated alongside outsourced 3D printing without a clear distinction. Our research also includes publications on the potential in-house use of 3D printing in maxillofacial surgery. To the best of our knowledge, this is one of the few studies that focuses only on point-of-care VSP and 3D printing in CMF surgery, addressing such a wide range of parameters over a long period of time (7 years). Additionally, this data collection is of great assistance when deciding to deploy a virtual surgical planning and 3D printing facility at the point-of-care.

Anatomical models are the most often produced in-house patient specific devices (39% of in-house CMF clinical applications) followed closely by cutting guides (32% of in-house CMF clinical applications), while molded cranial plates are the most often used in-house produced implanted devices. The direct printing of implantable devices requires costly equipment and particular circumstances that are difficult to obtain in a hospital environment; however, efforts have been made to develop techniques and printers that can solve this problem [66,71].

Planning surgery involves the use of dedicated software. Most software solutions come with paid licenses alongside the benefit of a user-friendly interface, easy learning curve, customer support, and medical certification. Due to their availability and cost-efficiency, many researchers/clinicians have turned toward free/open-source software solutions. In the European Union, virtual surgical planning software is defined as a medical device, and the use of a medically unauthorized software makes the surgeon accountable for a potential software-induced medical error. Nonetheless, the surgeon is equally accountable even if he/she uses a medically approved surgical planning software [43]. In the end, the Hippocratic principle of “primum non nocere” (“above all, do no harm“) is more prevalent than ever.

Printers that use FDM/FFF technology are by far the most used at the point-of-care, most likely due to their low-price tag and because of printing affordable anatomical models comparable with professional-grade models [28]. The main drawback of these printers is that they cannot print implantable devices yet, but efforts have been made to introduce desktop printers that can utilize Polyether Ether Ketone (PEEK) capable of printing patient-specific implants directly at the point-of-care, under the supervision of the treating surgeon [66,71]. SLA printers follow FDM printers in frequency of use, being widely exploited to print a broad spectrum of patient-specific devices like molds, cutting guides, or orbital models [3,34,51,56,59,60,61,62,65]. Finally, data found in the reviewed papers showed that hospital based 3DP laboratories access professional services when they need parts to be printed by selective laser sintering/melting (SLS/SLM) due to special conditions for printing and regulations.

According to our results, the primary human resource participating in the process of point-of-care 3D planning and printing include surgeons, radiologists, and information technology/bioengineering experts. Surgeons are increasingly enthusiastic about autonomy in virtual surgical planning and 3D printing to save costs, eliminate recurring online meetings, and prevent long delivery timeframes. Data regarding who is responsible for computer purchases, software, consumable materials, or maintenance were not addressed in the research reviewed. The imaging department and the 3D printing laboratory need to work together because the image datasets used in the digital workflow determine the end product’s quality and accuracy. The authors noticed a tendency for synergistic collaboration between the two entities. Establishing 3D printing laboratories inside or in strong collaboration with the radiology department is an example of good practice [6,18,26,34].

Concerning the timeline, this paper referred to three important parameters: the planning period, the printing and post-processing period, and the operation room’s time efficiency. The average planning time reported in the review-selected studies was 4.4 h. Because healthcare employees are paid by the hour and operating room cost-efficiency is assessed as money per unit of time, all these parameters also have an economic influence. The cost of planning time was not quantified, except for three studies [9,20,39], which is an evident flaw that must be addressed, as it highlights the issue of balancing time invested in planning and real economic/clinical benefits.

One of the key benefits of point-of-care 3D printing is that production takes less time than typical commercial delivery time frames [3,20,40,42,60]. Time management primarily impacts the outcome of patients suffering from acute afflictions, such as trauma or malignancy. Moreover, operation room time affects the patient’s clinical outcome in terms of the amount of time spent under general anesthesia, and it is also helpful in assessing indirect hospital cost savings due to the lessened use of the operation room [30]. While operative time can be shortened [6,18,25,30,35,42], this hypothesis requires prospective studies.

The cost of in-house 3D printing in CMF surgery can be perceived as high when summing up the initial investment in infrastructure but can also be considered low, when considering that a cutting guide is reported to cost 2 USD [44]. The reported costs of self-printed parts lack consistency. Most authors did not mention or only partially addressed direct costs (accommodation of infrastructure, software, training, computer, printer, and material costs) or time costs for 3D planning, printer set-up, post-processing, and maintenance.

Researchers must find ways to prove indirect savings obtained through operating room time economy. Studies must evaluate whether the externalization of VSP and 3DP means supplementary expense while the internalization of these services means savings for the hospital. POC 3DP promoters should also face central governmental authorities with research data pleading for an accelerated patient recovery leading to the immediate socio-economical reintegration of the patients that would otherwise be a burden for the social care system. Therefore, we encourage future research to present data in a much more structured, transparent, and objective manner, respecting health economics evaluation/reporting standards [77].

A consensus on reporting the surgical outcome of POC 3DP in CMF surgery could not be identified. Most of the studies reported positive outcomes but few provided quantitative evidence to support their clinical outcome. Consequently, neither of the selected studies measured surgical outcomes comprehensively. As point-of-care 3D printing becomes more mature, hospitals and clinics have started moving from simple applications to more complex applications, such as self-printed implantable devices. This process demands the demonstration of clinical efficacy and device safety. Consequently, to avoid researcher bias, the next important step is to involve as many independent groups of researchers as possible in validating POC-printed patient specific devices (PSI) through prospective clinical studies [78].

### 4.1. Limitations and Strengths

Despite its narrative nature, the current study provides a systematic and comprehensive overview on the concept of hospital-based virtual surgical planning and 3D printing. However, we are aware that some papers might have been missed. A lack of consistent data and heterogenous reports that are not always backed up by numbers and statistics suggest the need for more transparent and objective studies based on standardized reporting. Nevertheless, this is one of the first studies to address the use of in-house 3D printing in CMF surgery from such a broad time perspective—a span of seven years.

The results presented in this paper give an elaborate overview of the reported data on infrastructure, human resource, software, and printers used at the point-of-care. This set of data is highly valuable for anyone considering implementation/usage of virtual surgical planning and 3D printing at the point-of-care, not only in CMF surgery but also in other surgical specialties.

### 4.2. Further Research

Our review identified gaps that further research can fill: (1) a standardized guide to reporting data on the use of point-of-care 3D printing, applied not only to oral and cranio-maxillofacial surgery but also to the entire medical field; (2) a guide on the process of the integration of 3D printing and digital workflows in the hospital environment; and (3) the study of regulations and standards in order to establish verification and validation protocols, focused on monitoring point-of-care production processes with checkpoints to ensure device safety.

## 5. Conclusions

Oral and cranio-maxillofacial surgery supports the development of in-house 3D printed devices with promising results that suggest that the technology has reached maturity. The field of clinical applications is broad and continuously expanding, as it is currently being used from basic clinical applications up to complex surgical challenges. This data collection can help inform decisions when implementing virtual surgical planning and 3D printing in hospital departments or to serve as motivation for future research that can further develop point-of-care 3D printing in CMF surgery. In order to consolidate the role of point-of-care 3D printed devices in standard clinical practice and be seen as a viable alternative to outsourced professional solutions, further prospective, rigorous, and long-term assessments of clinical efficacy, cost-effectiveness, and device safety need to be conducted.

## Data Availability

Data are contained within the article. Additional information can be obtained from the corresponding author upon reasonable request.

## Supplementary Materials

The following supporting information can be downloaded at: https://www.mdpi.com/article/10.3390/jcm11226625/s1, Supplementary Table S1. Search strategies for PubMed database; Supplementary Table S2. Data collection process.
