# Supplementary material for: Point-of-Care Virtual Surgical Planning and 3D Printing in Oral and Cranio-Maxillofacial Surgery: A Narrative Review
Source: J Clin Med. 2022 Nov 8;11(22):6625. doi: 10.3390/jcm11226625 (PMC9692897; doi:10.3390/jcm11226625)
Supplement: Supplementary file 1 [file jcm-11-06625-s001.zip › jcm-1964976-supplementary.pdf]

Supplementary File

Supplementary Table S1. Search strategies for PubMed database.

Search Number	Keywords Association
1.	“3D printing” AND “maxillofacial surgery” AND “in-house”<br>(("printing, three dimensional"[MeSH Terms] OR ("printing"[All Fields] AND "three dimensional"[All Fields]) OR "three-dimensional printing"[All Fields] OR ("3d"[All Fields] AND "printing"[All Fields]) OR "3d printing"[All Fields]) AND ("surgery, oral"[MeSH Terms] OR ("surgery"[All Fields] AND "oral"[All Fields]) OR "oral surgery"[All Fields] OR ("maxillofacial"[All Fields] AND "surgery"[All Fields]) OR "maxillofacial surgery"[All Fields]) AND "in-house"[All Fields]) AND ((fft[Filter]) AND (humans[Filter]) AND (2015/1/1:2022/2/28[pdat]) AND (english[Filter]))
2.	“3D printing” AND “cranial surgery” AND “in-house”<br>(("printing, three dimensional"[MeSH Terms] OR ("printing"[All Fields] AND "three dimensional"[All Fields]) OR "three-dimensional printing"[All Fields] OR ("3d"[All Fields] AND "printing"[All Fields]) OR "3d printing"[All Fields]) AND (("cranially"[All Fields] OR "skull"[MeSH Terms] OR "skull"[All Fields] OR "cranial"[All Fields]) AND ("surgery"[MeSH Subheading] OR "surgery"[All Fields] OR "surgical procedures, operative"[MeSH Terms] OR ("surgical"[All Fields] AND "procedures"[All Fields] AND "operative"[All Fields]) OR "operative surgical procedures"[All Fields] OR "general surgery"[MeSH Terms] OR ("general"[All Fields] AND "surgery"[All Fields]) OR "general surgery"[All Fields] OR "surgery s"[All Fields] OR "surgerys"[All Fields] OR "surgeries"[All Fields])) AND "in-house"[All Fields]) AND ((fft[Filter]) AND (humans[Filter]) AND (2015/1/1:2022/2/28[pdat]) AND (english[Filter]))
3.	“3D printing” AND “maxillofacial surgery” AND “hospital printed”<br>(("printing, three dimensional"[MeSH Terms] OR ("printing"[All Fields] AND "three dimensional"[All Fields]) OR "three-dimensional printing"[All Fields] OR ("3d"[All Fields] AND "printing"[All Fields]) OR "3d printing"[All Fields]) AND ("surgery, oral"[MeSH Terms] OR ("surgery"[All Fields] AND "oral"[All Fields]) OR "oral surgery"[All Fields] OR ("maxillofacial"[All Fields] AND "surgery"[All Fields]) OR "maxillofacial surgery"[All Fields]) AND (("hospital s"[All Fields] OR "hospitalisation"[All Fields] OR "hospitalization"[MeSH Terms] OR "hospitalization"[All Fields] OR "hospitalising"[All Fields] OR "hospitality"[All Fields] OR "hospitalisations"[All Fields] OR "hospitalised"[All Fields] OR "hospitalizations"[All Fields] OR "hospitalized"[All Fields] OR "hospitalize"[All Fields] OR "hospitalizing"[All Fields] OR "hospitals"[MeSH Terms] OR "hospitals"[All Fields] OR "hospital"[All Fields]) AND ("printed"[All Fields] OR "printing"[MeSH Terms] OR "printing"[All Fields] OR "print"[All Fields] OR "printings"[All Fields] OR "prints"[All Fields]))) AND ((fft[Filter]) AND (humans[Filter]) AND (2015/1/1:2022/2/28[pdat]) AND (english[Filter]))
4.	“3D printing” AND “cranial surgery” AND “hospital printed”<br>(("printing, three dimensional"[MeSH Terms] OR ("printing"[All Fields] AND "three dimensional"[All Fields]) OR "three-dimensional printing"[All Fields] OR ("3d"[All Fields] AND "printing"[All Fields]) OR "3d printing"[All Fields]) AND (("cranially"[All Fields] OR "skull"[MeSH Terms] OR "skull"[All Fields] OR "cranial"[All Fields]) AND ("surgery"[MeSH Subheading] OR "surgery"[All Fields] OR "surgical procedures, operative"[MeSH Terms] OR ("surgical"[All Fields] AND "procedures"[All Fields] AND "operative"[All Fields]) OR "operative surgical procedures"[All Fields] OR "general surgery"[MeSH Terms] OR

	("general"[All Fields] AND "surgery"[All Fields]) OR "general surgery"[All Fields] OR "surgery s"[All Fields] OR "surgerys"[All Fields] OR "surgeries"[All Fields])) AND (("hospital s"[All Fields] OR "hospitalisation"[All Fields] OR "hospitalization"[MeSH Terms] OR "hospitalization"[All Fields] OR "hospitalising"[All Fields] OR "hospitality"[All Fields] OR "hospitalisations"[All Fields] OR "hospitalised"[All Fields] OR "hospitalizations"[All Fields] OR "hospitalized"[All Fields] OR "hospitalize"[All Fields] OR "hospitalizing"[All Fields] OR "hospitals"[MeSH Terms] OR "hospitals"[All Fields] OR "hospital"[All Fields]) AND ("printed"[All Fields] OR "printing"[MeSH Terms] OR "printing"[All Fields] OR "print"[All Fields] OR "printings"[All Fields] OR "prints"[All Fields]))) AND ((fft[Filter]) AND (humans[Filter]) AND (2015/1/1:2022/2/28[pdat]) AND (english[Filter]))
5.	“three-dimensional printing” AND “maxillofacial surgery” AND “in-house” (("printing, three dimensional"[MeSH Terms] OR ("printing"[All Fields] AND "three dimensional"[All Fields]) OR "three-dimensional printing"[All Fields] OR ("three"[All Fields] AND "dimensional"[All Fields] AND "printing"[All Fields]) OR "three dimensional printing"[All Fields]) AND ("surgery, oral"[MeSH Terms] OR ("surgery"[All Fields] AND "oral"[All Fields]) OR "oral surgery"[All Fields] OR ("maxillofacial"[All Fields] AND "surgery"[All Fields]) OR "maxillofacial surgery"[All Fields]) AND "in-house"[All Fields]) AND ((fft[Filter]) AND (humans[Filter]) AND (2015/1/1:2022/2/28[pdat]) AND (english[Filter]))
6.	“three-dimensional printing” AND “cranial surgery” AND “in-house” (("printing, three dimensional"[MeSH Terms] OR ("printing"[All Fields] AND "three dimensional"[All Fields]) OR "three-dimensional printing"[All Fields] OR ("three"[All Fields] AND "dimensional"[All Fields] AND "printing"[All Fields]) OR "three dimensional printing"[All Fields]) AND (("cranially"[All Fields] OR "skull"[MeSH Terms] OR "skull"[All Fields] OR "cranial"[All Fields]) AND ("surgery"[MeSH Subheading] OR "surgery"[All Fields] OR "surgical procedures, operative"[MeSH Terms] OR ("surgical"[All Fields] AND "procedures"[All Fields] AND "operative"[All Fields]) OR "operative surgical procedures"[All Fields] OR "general surgery"[MeSH Terms] OR ("general"[All Fields] AND "surgery"[All Fields]) OR "general surgery"[All Fields] OR "surgery s"[All Fields] OR "surgerys"[All Fields] OR "surgeries"[All Fields])) AND "in-house"[All Fields]) AND ((fft[Filter]) AND (humans[Filter]) AND (2015/1/1:2022/2/28[pdat]) AND (english[Filter]))
7.	“three-dimensional printing” AND “maxillofacial surgery” AND “hospital printed” (("printing, three dimensional"[MeSH Terms] OR ("printing"[All Fields] AND "three dimensional"[All Fields]) OR "three-dimensional printing"[All Fields] OR ("three"[All Fields] AND "dimensional"[All Fields] AND "printing"[All Fields]) OR "three dimensional printing"[All Fields]) AND ("surgery, oral"[MeSH Terms] OR ("surgery"[All Fields] AND "oral"[All Fields]) OR "oral surgery"[All Fields] OR ("maxillofacial"[All Fields] AND "surgery"[All Fields]) OR "maxillofacial surgery"[All Fields]) AND (("hospital s"[All Fields] OR "hospitalisation"[All Fields] OR "hospitalization"[MeSH Terms] OR "hospitalization"[All Fields] OR "hospitalising"[All Fields] OR "hospitality"[All Fields] OR "hospitalisations"[All Fields] OR "hospitalised"[All Fields] OR "hospitalizations"[All Fields] OR "hospitalized"[All Fields] OR "hospitalize"[All Fields] OR "hospitalizing"[All Fields] OR "hospitals"[MeSH Terms] OR "hospitals"[All Fields] OR "hospital"[All Fields]) AND ("printed"[All Fields] OR "printing"[MeSH Terms] OR "printing"[All Fields] OR "print"[All Fields] OR "printings"[All Fields] OR "prints"[All Fields]))) AND ((fft[Filter]) AND (humans[Filter]) AND (2015/1/1:2022/2/28[pdat]) AND (english[Filter]))
8.	“three-dimensional printing” AND “cranial surgery” AND “hospital printed” (("printing, three dimensional"[MeSH Terms] OR ("printing"[All Fields] AND "three dimensional"[All Fields]) OR "three-dimensional printing"[All Fields] OR ("three"[All Fields] AND "dimensional"[All Fields] AND "printing"[All Fields]) OR "three dimensional printing"[All Fields]) AND (("cranially"[All Fields] OR "skull"[MeSH Terms] OR "skull"[All

	Fields] OR "cranial"[All Fields]) AND ("surgery"[MeSH Subheading] OR "surgery"[All Fields] OR "surgical procedures, operative"[MeSH Terms] OR ("surgical"[All Fields] AND "procedures"[All Fields] AND "operative"[All Fields]) OR "operative surgical procedures"[All Fields] OR "general surgery"[MeSH Terms] OR ("general"[All Fields] AND "surgery"[All Fields]) OR "general surgery"[All Fields] OR "surgery s"[All Fields] OR "surgerys"[All Fields] OR "surgeries"[All Fields])) AND (("hospital s"[All Fields] OR "hospitalisation"[All Fields] OR "hospitalization"[MeSH Terms] OR "hospitalization"[All Fields] OR "hospitalising"[All Fields] OR "hospitality"[All Fields] OR "hospitalisations"[All Fields] OR "hospitalised"[All Fields] OR "hospitalizations"[All Fields] OR "hospitalized"[All Fields] OR "hospitalize"[All Fields] OR "hospitalizing"[All Fields] OR "hospitals"[MeSH Terms] OR "hospitals"[All Fields] OR "hospital"[All Fields]) AND ("printed"[All Fields] OR "printing"[MeSH Terms] OR "printing"[All Fields] OR "print"[All Fields] OR "printings"[All Fields] OR "prints"[All Fields]))) AND ((fft[Filter]) AND (humans[Filter]) AND (2015/1/1:2022/2/28[pdat]) AND (english[Filter]))
9.	“additive manufacturing” AND “maxillofacial surgery” AND “in-house” (("addit manuf"[Journal] OR ("additive"[All Fields] AND "manufacturing"[All Fields]) OR "additive manufacturing"[All Fields]) AND ("surgery, oral"[MeSH Terms] OR ("surgery"[All Fields] AND "oral"[All Fields]) OR "oral surgery"[All Fields] OR ("maxillofacial"[All Fields] AND "surgery"[All Fields]) OR "maxillofacial surgery"[All Fields]) AND "in-house"[All Fields]) AND ((fft[Filter]) AND (humans[Filter]) AND (2015/1/1:2022/2/28[pdat]) AND (english[Filter]))
10	“additive manufacturing” AND “cranial surgery” AND “in-house” (("addit manuf"[Journal] OR ("additive"[All Fields] AND "manufacturing"[All Fields]) OR "additive manufacturing"[All Fields]) AND (("cranially"[All Fields] OR "skull"[MeSH Terms] OR "skull"[All Fields] OR "cranial"[All Fields]) AND ("surgery"[MeSH Subheading] OR "surgery"[All Fields] OR "surgical procedures, operative"[MeSH Terms] OR ("surgical"[All Fields] AND "procedures"[All Fields] AND "operative"[All Fields]) OR "operative surgical procedures"[All Fields] OR "general surgery"[MeSH Terms] OR ("general"[All Fields] AND "surgery"[All Fields]) OR "general surgery"[All Fields] OR "surgery s"[All Fields] OR "surgerys"[All Fields] OR "surgeries"[All Fields])) AND "in-house"[All Fields]) AND ((fft[Filter]) AND (humans[Filter]) AND (2015/1/1:2022/2/28[pdat]) AND (english[Filter]))
11.	“additive manufacturing” AND “maxillofacial surgery” AND “hospital printed” (("addit manuf"[Journal] OR ("additive"[All Fields] AND "manufacturing"[All Fields]) OR "additive manufacturing"[All Fields]) AND ("surgery, oral"[MeSH Terms] OR ("surgery"[All Fields] AND "oral"[All Fields]) OR "oral surgery"[All Fields] OR ("maxillofacial"[All Fields] AND "surgery"[All Fields]) OR "maxillofacial surgery"[All Fields]) AND (("hospital s"[All Fields] OR "hospitalisation"[All Fields] OR "hospitalization"[MeSH Terms] OR "hospitalization"[All Fields] OR "hospitalising"[All Fields] OR "hospitality"[All Fields] OR "hospitalisations"[All Fields] OR "hospitalised"[All Fields] OR "hospitalizations"[All Fields] OR "hospitalized"[All Fields] OR "hospitalize"[All Fields] OR "hospitalizing"[All Fields] OR "hospitals"[MeSH Terms] OR "hospitals"[All Fields] OR "hospital"[All Fields]) AND ("printed"[All Fields] OR "printing"[MeSH Terms] OR "printing"[All Fields] OR "print"[All Fields] OR "printings"[All Fields] OR "prints"[All Fields]))) AND ((fft[Filter]) AND (humans[Filter]) AND (2015/1/1:2022/2/28[pdat]) AND (english[Filter]))
12.	“additive manufacturing” AND “cranial surgery” AND “hospital **p**rinted” (("addit manuf"[Journal] OR ("additive"[All Fields] AND "manufacturing"[All Fields]) OR "additive manufacturing"[All Fields]) AND (("cranially"[All Fields] OR "skull"[MeSH Terms] OR "skull"[All Fields] OR "cranial"[All Fields]) AND ("surgery"[MeSH Subheading] OR "surgery"[All Fields] OR "surgical procedures, operative"[MeSH Terms] OR ("surgical"[All Fields] AND "procedures"[All Fields] AND "operative"[All Fields]) OR "operative surgical

	procedures"[All Fields] OR "general surgery"[MeSH Terms] OR ("general"[All Fields] AND "surgery"[All Fields]) OR "general surgery"[All Fields] OR "surgery s"[All Fields] OR "surgerys"[All Fields] OR "surgeries"[All Fields])) AND (("hospital s"[All Fields] OR "hospitalisation"[All Fields] OR "hospitalization"[MeSH Terms] OR "hospitalization"[All Fields] OR "hospitalising"[All Fields] OR "hospitality"[All Fields] OR "hospitalisations"[All Fields] OR "hospitalised"[All Fields] OR "hospitalizations"[All Fields] OR "hospitalized"[All Fields] OR "hospitalize"[All Fields] OR "hospitalizing"[All Fields] OR "hospitals"[MeSH Terms] OR "hospitals"[All Fields] OR "hospital"[All Fields]) AND ("printed"[All Fields] OR "printing"[MeSH Terms] OR "printing"[All Fields] OR "print"[All Fields] OR "printings"[All Fields] OR "prints"[All Fields]))) AND ((fft[Filter]) AND (humans[Filter]) AND (2015/1/1:2022/2/28[pdat]) AND (english[Filter]))

Supplementary Table S2. Data collection process.

Author and Year of Publication	Applications	Accommodation of Infrastructure	Human Resource	Software	Hardware-3D Printers and Materials	Planning Time	Production- 3D Printing Time	Operating Room Time	Cost	Outcome
Mendez et al., 2015 [17]	Skull model	In-house (In office)	Surgeon	Rapid3D – OS	MakerBot Replicator-FDM	NA	14 h	Case 1/2: 4.5h/4h OR time	P: 2750$ (Printer protection plan: 350$/year). M: 25$/model Training by Maker Bot: 225$; Additional cost: physician's time and effort-NA	Cost-effective (Outsourced models costing 4000$). Less time-consuming Efficient method of creating surgical models -
Weinstock et al. 2015 [18]	Models of AVM	Hospital lab ran by Boston Children's hospital Simulator Program	Neuroradiolo gist and treating clinician	NA	Connex 500 (Stratasys)-PolyJet	2–6 hours of imaging review and model segmentation	6–12 h	OR time: 30 min. faster with 3D model	NA	30 minutes faster OR time with 3D models. Neurovascular lesions are printable in less than 24 hours ;(3) direct correlation between 3D models and surgical anatomy
Matsumoto et al. 2015 [37]	Models (Anatomy, education, research, forensics, and quality control)	In-house (Radiology department)	Technologist + radiologist / radiologist alone/ radiologist and surgeon	MIMICS, 3-matic- L	Objet350 Connex; Stratasys-PolyJet; Dimension Elite Fortus 400mc-FFF	NA	A few hours to multiple days (no numbers given)	NA	M: few dollars to several thousand dollars (no numbers given)	Safety, efficiency, and reduction in OR time. Increased surgeon confidence in a positive outcome.
Mottini et al. 2016 [19]	Models (For pre-bending), Fibula cutting guide	Not clearly stated but with in-house potential use evoked	surgeon	Amira- L Blender-OS	P:-NA M: Med 610 photopolymer	NA	Less than 48h	Prebent plate reduces operative time	NA	Guide offers better stability precision
Ahmed et al. 2016 [22]	Temporal bone models	Not clearly stated but with in-house potential use evoked	NA	Mimics-L	P: undisclosed –FDM M: PLA, ABS	NA	NA	Surgeon noticed decreased time and ease of	P: 1,200$; M: < 5$/ PLA model, <5$/ABS model	Models compared to tegmen anatomy based upon imaging and IO findings

								graft placement-no time data to validate this notion.		
Goetze et al.2017 [39]	Fibula cutting guide	Not clearly stated but with in-house potential use evoked	Surgical resident	Simplant O&O®-L OsiriX-L NetFabb Basic-L(free) MeshLabOS PlastyCAD®-L	Stratasys EDEN260V PolyJet. M: MED610 photopolymer	First plannings took 10 h while the last plannings took around 5 h	Planning was performed in 4 days (text suggests planning and printing)	Osteotomy and assembly time did not exert 1 h in all cases	(1) in-house workflow (517–863€) (2) Self-printed 3D model 150–220€/ planning (3) Material for a skull and fibula with 220€ *Without printer/software costs	Involvement of surgeons in planning might have a positive effect on flap survival and complication rate.
Legocki et al. 2017 [20]	Pediatric mandible models	IT department of the medical center.	IT staff member	OsiriX-L MeshLab-OS	MakerBot Replicator 3D printer-FDM	20 to 60 min. for software processing and printer preparation	Print time 6-12 h Postprocessing: 1h Protocol resulted in a next-day model, faster than commercial by at least 1day	NA	P: 3D 2899$ -OsiriX: $699/year -PLA $50/kg -IT hospital employee $45/ h	The cost: 3-D printing technology and software pays for itself after 4 prints
Msallem et al. 2017 [72]	Cranial implant analogue for molding	Not clearly stated but with in-house potential use evoked	NA	MIMICS-L 3-matic-L DDS-Pro-L	MakerBot Replicator 3D Printer (5th Gen.)-FDM	NA	Printing;1-5 h (palm-size templates)	NA	DDS-Pro –300$ (lifelong L)	Reduction of blood loss, OR time Reduced general anesthesia time=>costs decrease, reduced comorbidity.
Abdel Hay et al.2017 [57]	Mold for cranial plate	Not clearly stated but with in-house potential use evoked	NA	Horos, MeshMixer, Blender-OS/free	Delta2040-FDM	mold design-2h	Print time: 2h56m - 9h 6m	Patient1- 1h; Patient 2- 1h and 25m	Printer-5000$; PLA/mold- 5$; Cost/ implant-230$	Cranial implant fitted precisely into the defect
Liu et al.2017 [73]	Cerebral aneurysm simulator	Not clearly stated but with in-house potential use evoked	NA	Mimics, Magics-L	P: D-Force 400; M: PLA, PVA, silicone	NA	NA	NA	P:600$; M: PLA 11.90$/kg; PVA 21.70$/kg Simulator 22.88$	Facilitated the training. Better 3D understanding aneurysm. Allows creation of multiple medical scenarios

Callahan et al.2017 [63]	Orbital implant templates used as stencil and mold for actual implant	Hospital affiliated institutions	NA	Vitrea, Mimics,3-Matic-L	P: Makerbot Replicator 2, Stratasys Fortus 250mc-FDM<br>M: ABS plus	NA	NA	NA	NA	10.6 months follow up-no complications and no further revision
Velasco et al.2017 [21]	Mandibular models printed for planning and plate prebending	Not clearly stated but with in-house potential use evoked	NA	InVesalius, Meshmixer, MatterControl-OS/free	P: ROBO 3D R1-FDM<br>M: PLA	75 min. (segmentation, isolation, and fix)	Print time: - 4-7 hours depending on detail and size (Case 1-5h 31m, Case 2-4h 38m Case 3- 5h 20m)	OR time: Case 1- 3h 50m, Case 2 - 5h 30m, Case 3- 5h 15m. Suggested time savings 1 or 2 hours (based on literature data)	P: $ 1,000;<br>M: PLA – 0.022$<br>Cost/model: Case1-0.9$, Case 2- 0.67$, Case3 - 0.99$(model weight in g)	Precise models: Plates fixed without IO modification.<br>PO symmetry.<br>Reduced OR time, costs, patient morbidity, anesthesia risk.<br>Increased patient satisfaction.
Bosc et al.2017 [40]	Mandibular/fibula cutting guides	In-house	Surgeon	Ayra (undisclosed/ (unavailable data on license)<br>, OsiriX-L<br>3D Slicer, Meshmixer, Blender-OS	Stratasys Objet30 Pro-PolyJet;<br>Zortrax M200-FDM	Time required to produce the virtual model- 12 to 48 h.	Fabrication process; 3-15 days (mean 5.1 days);<br>Could be reduced to 48 h.	OR time: 6 to 10 h (mean 7 h 2 m)	Additional cost: 17,800E (3D printers, software, consumables) => 989E /patient, 195E cutting guide;<br>P: Zortrax M200-1800 E	All mandibular reconstructions were successful with a good match between the PO 3D planning and the results.<br>Simulated bone segments<br>and angles similar to PO volume renderings (P =0.61).
Hatamleh et al.2017 [41]	Mandibular hyperplasia cutting guides	Not clear stated but with in-house purpose of use evoked	NA	CMF Pro Plan, 3-Matic -L	P: undisclosed;<br>M: Model 610 biocompatible resin	NA	NA	Patient 1: Surgery lasted for 70 min.;<br>Patient 2: undisclosed	NA	3D technologies are accurate and reliable in the designing of cutting guides for the correction of hemi mandibular hyperplasia in timely and cost-effect manner
Ganry et al.2017 [42]	Fibula cutting guide	In-house: planning;<br>Outsourced: Printing.	Surgeon	Osirix-L, MeshLab, Netfabb,<br>and Blender-OS	P: undisclosed (SLS);<br>M: polyamide 12 (poly-laurolactam)	NA	Time frame for printing, delivery, and sterilization was less than 7 days.	Surgical procedure time was reduced by 1.5 h	Overall cost was < 100 E/case (due to object printing) - vs. several thousand<br>E in case of commercial service),	Enhanced flap viability;<br>Reduced risk of neoplastic cell contamination;<br>Precision in line with precision obtained using specialized companies.

Ganry, Hersant, et al. 2017 [43]	Fibula cutting guide	In-house (hospital production suggested)	Surgeon	OsiriX, Netfabb -L Meshlab, Blender - OS	NA	NA	NA	NA	Even with 3D printing professional assistance, the costs would not exceed 25E/fibular and 8E/ Mandibular SG	To recommend this protocol as an alternative to professional service, further studies are needed to determine if accuracy is similar to the professional technologies.
Werz et al.2017 [75]	Surgical Simulator (Upper and lower jaw)	Laboratory work but validated for in-house use	Maxillofacial surgeons	3D Slicer, Blender- OS	P: MakerBot Replicator 5th Generation; M: ABS or PLA	NA	4 models printed simultaneously in 4.5h (mandible),5.5h(maxilla)	NA	model cost between 1.40-1.60$ (ABS) and 1.80-2.00$ (PLA)	3D printing with inexpensive FDM 3D printers is a promising method to create training models
Smithers et al. 2017 [46]	Cutting guides (Mandibular and fibula)	In-house	Surgical team technician – both on same physical site.	3D Slicer, Instant Mesh, Mesh lab – OS 3DS Max, Z Brush - L	P: UP BOX Printer- FDM	Acquisition phase (6 h); Planning phase (16 h)	Printing time: mandibular/fibula guides, external fixator: 8 h; Post processing:2h	Median operative time was 7 h 46 min (range: 6 h 50 min–9 h 51 min)	P: printer-4000$; M: PLA-60$/0.5kg Software: 3DS Max-6195$/3 y	VSP - accurate, reduce OR time and less stressful for surgeons (based on references, authors did not measure these outcomes)
Numajiri, Morita, Nakamura, Tsujiko, et al. 2017 [47]	Maxillary and fibula cutting guides	In-house	Surgeon	Blender-OS	P: MakerBot Replicator 3D printer 5th generation	4.1 h for PO planning	12 h for guide printing	Average OR time was 17 h 21 min. (ischemic time: flap suturing/bone fixation, 70 min., anastomoses, 64 min.)	P: 4000$ The in-house approach is 2$/guide (but the cost is much higher when preparation, effort and surgeon’s time are included.	Mean deviation after reconstruction was 0.44 mm (standard deviation, 0.97 mm). Deviations were 67.8% for 1 mm, 93.8% for 2 mm, and 98.6% for 3 mm. All reconstructions were judged as accurate.
Numajiri et al.2018 [44]	Maxillary and fibula cutting guide	In-house (hospital production suggested)	Surgeons	InVesalius, Blender-OS	P: undisclosed-FDM; M: PLA	Surgeons do it in their spare time (no numbers)	NA	Time-saving (based on references, no clear numbers or statistics)	In-house approach seems cheaper than commercial approaches, the real cost (including	Accurate osteotomy length, width, and angle in cutting bones while using cutting guides. (Based on references, no clear numbers or statistics)

									surgeon's effort) is neglected.	
Dell'Aversana Orabona et al.2018 [45]	Mandibular and fibula cutting guides	In-house	Surgeons	InVesalius, 123D Design - OS	P: Ultimaker 2 - FDM<br>M: PLA	Virtual planning was about 3 h	Printing process and sterilization:6h	NA	P:3000 Euro.<br>M:6 meters of PLA - 3.6 E	An average mean distance of 1.631 mm (range 0.594 - 4.067) and a standard deviation of 5.496 mm (range 1.966 - 8.024) has been demonstrated 3D volumes overlay analysis.
Elegbede et al. 2018 [23]	Models of virtually reduced fractures (for plate bending)	In-house	Surgeon	3D Slicer – OS;<br>NetFabb Standard-L	P: LulzBot TAZ 6;<br>M: ABS	Image processing takes <30 minutes	2-12 h (complete mandible about 6 h)	OR time can be reduced (based on author's experience)	P: 2500$;<br>M: 43$/kg;<br>S: NetFabb Standard: 1000$/y	NA<br>(There have been no randomized studies showing better patient outcomes when this technology is applied)
Evins et al. 2018 [58]	FDM experimentally produced craniofacial prosthesis and molds	Laboratory work but validated for in-house use. (IO production - simulation of process on cadaveric models)	Surgeon (suggested)	Mimics; 3-matic -L	P: Stratasys Fortus 250mc-FDM;<br>M: ABS	Average time for design (CT import to print initiation was): 14.6 min.	Average print time for all cranioprostheses : 108.6min.;<br>At medium resolution- less than 3 h	NA	NA<br>Variable costs made impossible a comprehensive analysis.<br>Printed prostheses cost: < 1$ (using PMMA and designed by clinical personnel).	Seamlessly approximated the defect, followed skull curvature, were flush with the skull and easily fixed using conventional titanium gear (just qualitatively assessed)
Philipp Honigmann et al. 2018 [66]	FFF experimental production PSI	Research facility but tested and validated for in-house use.	Surgeon (suggested)	Mimics; 3-matic -L	P: Apium P220-FFF<br>M: PEEK	NA	NA		NA	NA
Valding et al. 2018 [24]	Orbital floor model	Research facility but tested for in-house use.	Technician and physician	Medical use: Mimics-L;<br>Not medically approved: InVesalius	P: Professional: 3D System ProJet 3510 - MJP;<br>Nonprofession al:	Professional: Mimics: plan time- 1 h 15 min to 1 h 30 min;	Professional: Mimics: total time- 22 h 30 min (18 h print time);<br>Nonprofessional:	NA	P: 3D System Projet 3510)- 50,000E;<br>FABtotum - 1300 E;	NA

				MeshMixer, Netfabb - OS	FABtotum Core-FDM	Nonprofessional (InVesalius, Meshmixer, Netfabb):1 h 50 min to 3 h 20 min	31 h 30 min (24h 30min print time: booting, machine setting, printing, cleaning t)		M: PLA—35E / 750 g Resin—570E/2 kg S: Mimics: 5000-12,000E/y; Nonprofessional -0 -5000 E/y	
Naros et al.2018 [25]	Mandible models for pre-bent reconstruction plates	In-house	NA	IPlan CMF 3.0-L	P: ProJet® CJP 360 - BJ	DICOM to .STL file-5 min	270 min (190 min powder bed and inkjet head 3D printing; 90 min dried and fixed with resin)	IO time saving of 30–45 min.	NA (Investment outweighs the reduced surgery costs (surgical team, anesthesia care team, materials)	Method prevents rotational error of the mandibular angle; improves restoration of intercondylar angle
McAllister et al. 2018 [49]	Cutting and positioning guide (Orthognathic Surgery)	In-house	Surgeon	3D slicer, Meshmixer-OS	P: printer undisclosed-FDM	NA	Production takes frequently < 3 days (including 36 h for model and guide printing, sterilization) NOTE: Also mentioned production typically takes < 24-h total print time	Custom guides reduce operative duration, anesthetic time - not backed up by numbers	P: printer undisclosed-1000£ M: 10£/case Outsourced models and guides for a bimaxillary osteotomy: 1500– 2000£	Increased surgical predictability; Reduced OR time, anesthetic time and theatre costs- not backed-up by numbers and statistics
Morales-Gómez et al.2019 [59]	Mold for PMMA cranioplasty	In-house (Confirmed by full text reading)	Surgeon (suggested)	InVesaliu, Blender, MeshLab, MeshMixer-OS	P: Formlabs Form 2-SLA; Ultimaker 2+-FDM	Design process: 4-5 h	3D mold printing: 10 h	OR ranged 90-150 min. (mean 126 min.)	P: Form 2 - 3499$); Ultimaker 2+ - 2499$; Implant ranged 135.23$ to 444.44$ (mean307.79$)	Excellent patient, family, and surgeon satisfaction at a fraction of the cost of commercial implants
Spaas and Lenssen 2019 [9]	Model of mandibular reconstruction with fibula	In-house	Surgeon	3D slicer-OS; Netfabb Basic-L/Free, iPlan 3.0.5 (Brainlab)-L	P: Leapfrog Creatr-FDM	18.5 (15-25) min. segmentation; 33.5 (18-53) min to plan the	266.5 (261-272) min. of 3D printing; Planning, printing, and	NA	Labour cost: 15.24E/h (junior surgeon); Additional cost/ patient	Protocol is: - cost-effective; - easy to use; - accurate - (sustained by numbers and statistics)

						fibular osteotomy	laboratory time total: 6 h		276 E (257.50-297.25E)	
Freiser et al. 2019 [67]	Temporal bone model (simulator)	In-house (Radiology-based printing lab)	Radiologist and print technicians	3D slicer, Meshmixer, Blender-OS	P: Formlabs Form 2	CT image upload to print: 3.1 h	Print time: 7.0 h/model; Postprocessing time: 1.5 h	NA	P: Form 2-4000$; M: resin cartridge 200$; UV box- 800$ $103.08/model	Model is an excellent utility for training purposes (median 9.3; range 8.4–9.9) and for reference during surgery (median 9.5, range 7.5–9.7);
Guest et al. 2019 [26]	Anatomical models	Radiology department 3D planning lab and joint engineering center (houses the printer)	Radiologist and surgeon	InVesalius - OS; Freeform, Design X, SOLIDWORKS-L	P: Stratasys uPrint SE Plus-FDM	Structure segmentation:1-4h	Print time: “several h”; Production: 4d (1d for DICOM processing and 3d of printing and assembly).	Just hypothesis: model availability could reduce OR time.	Average model cost: 350$ (250$ for polymer printing substrate and 100$ technical setup)	Models improved surgeon confidence. Just hypothesis: model availability could reduce OR time and risk of PO complications.
Marschall et al. 2019 [6]	Mandible model for plate pre-bending	In-house	Radiologist and surgeons	Mimics, 3-matic-L	P: New- Pro NP1(NewPro 3D prototype)-DLP; Alternative: Form2- SLA	Virtual fracture reduction: 20 min (depends on user’s software and anatomy familiarity	Print time: New-Pro NP1: 1 h; Alternative: Form2- 5-7 h Isopropyl alcohol wash and curing: 15 min. each.	OR time 1.5 h vs. 2.25 h for traditional ORIF	P: New- Pro NP1- price undisclosed; Alternative: Form2: 3500$ M: resin (VeriModel OS)-175$/kgReRsin tank-100$(10x use); Curing station: $500; Cost/model: 50$	Fit clinically identical to the virtually reduced model; No IO plate manipulation needed. Further studies needed to elucidate cost/case, OR time reduction, and accuracy
King et al. 2019 [27]	Mandible model for plate pre-bending	In-house	Surgeon	3D Slicer, Meshmixer-OS	NA	NA	NA	"Potential for reduced theatre time "-not backed up by numbers	“Expenditure of less than 100£” when “UK commercial printers charge in the region of 300-400£/ mandibular model.	Accurate fracture fixation. Reduced OR time makes investment worth despite additional training and initial expenditure

Abbate et al. 2019 [64]	Orbital floor mold for pressure bending of orbital mesh	In-house	Surgeon (suggested)	iPlan Cranial 3.0-L Meshmixer, 123D Design-OS	P: Ultimaker 2-FDM; M: PLA	Time spent on planning lead to consistent gain during surgery. - not backed-up by numbers	Assumed to take 24 hours but not backed up by numbers	NA	3 meters of filament/3 E	Good superimposition of preoperative planning over the PO result in all the cases - backed-up by numbers and statistics
Yang et al. 2019 [4]	Cutting guides, osteosynthesis plate	In-house (cutting guides- design and printed; plate- design)	Surgeon: cutting guides, plate prototype; Engineer: optimized prototype	ProPlan CMF 3.0, 3-matic 13.0 -L	Outsourced P: SLM125 –SLM; In-house P: P undisclosed M: grade 2 Ti, ULTEM 1010, MED610 resin	Virtual surgery and plate design: 18.83 +/- 13.19 h	Plate optimization, printing, post-processing, delivery: 162.9 +/-55.15h (4-9 d)	NA	NA	Superimposing PO model onto the virtual plan -> mean absolute distance deviation 0.89 +/- 1.02 mm => optimal accuracy
Hatz CR, et al. 2019 [28]	Mandibular models (printed both inhouse and outsourced for accuracy evaluation)	In-house (+ external facility)	Surgeon (suggested)	3-matic-L	P: In-house: MakerBot Replicator+-FDM; External facility: EOSINT P 385-SLS; M: PLA, Polyamide 12	In-house fabrication takes few h (digital preparation and print) - not backed-up by numbers	In-house fabrication takes few h (digital preparation and print) - not backed-up by numbers	NA	P: MakerBot Replicator +- 2499$; EOSINT P 385 –300.000$ M: PLS- 48$/0.9kg Inhouse vs. commercial model: 4.46$ vs. 664$ Excluded: personnel, software L	In-house models are accurate and comparable to professional models - backed-up by numbers and statistics => therefore equally suitable for clinical work
Abo Sharkh and Makhoul 2019 [3]	Mandible, fibula, scapula models and cutting guides	In-house	Surgeon	3DSlicer, Meshmixer-OS	P: Qidi PLA-FDM; Form 2-SLA	Total VSP: 158 min (2h 38m)- segmentation (14 min.), VSP (99min.), virtual cutting guides (46 min)	Printing time: Cutting guides 2h 4min. (SLA); Models:5h 30min. In house protocol takes ,24h vs. 2-4 weeks in case of third-party VSP	NA	P: Form 2- 5000$ CAD; Qidi PLA- 900$ CAD; Surgical model- 5.21$ CAD; Cutting guides$ 12.80 CAD) - based material and amortized equipment	Cost efficiency
Zavattero et al. 2020 [48]	Mandibular and fibula cutting guides	In-house	Surgeon	3D Slicer, Blender, Meshmixer-OS SolidWorks-L	P: Ultimaker-FDM	Average designing time for the guides: 4.6 h	Print time: one guide 4 h (depending on volume)	3D models and cutting guides help reduce the OR time	P: Ultimaker- 5000$;	Accurate method; Reliable 3D printing in a hospital 3D lab;

									M: material/guide -10$; Cost of designing- 0$(medical doctors)	Anatomical models and guides feasible for daily clinical use at reasonable costs.
Chamo et al. 2020 [74]	Cranioplasty templates for in-house fabrication of cranial plates	Not clear stated but with in-house purpose of use evoked	Surgeon	MIMICS, 3-matic-L	P: MakerBot Replicator+; M: PLA	NA	NA	NA	NA	Molded PMMA implants based on 3D printed templates is highly precise- < 1 mm deviation evaluated in two different defects
Hassan et al. 2020 [29]	Mandibular model and surgical template(guide)	In-house	Surgeon	3D Slicer R, Meshmixer-OS	P: Flashforge R Creator Pro-FDM<br>M: PLA	Segmentation time of the CT scan to VP: Case 1 (2 h 35 min), Case 2 (1 h 15 min) and Case 3 (45 min)	Print time: Case 1 (4 h 29 min), Case 2 (3 h 27 min) Case 3 (3 h 55min); Segmentation ->VP ->3D printing- Average/case: 5 h 29 min	Technique is reported to reduce OR time and increase accuracy-not backed up by numbers	P: Flashforge Creator Pro- 915$;<br>M: PLA- 20.29$/kg;<br>Labor: 4.57$ h<br>Average/case: 203.42, if excluding printer cost average cost/case: 10.35$	Reduced OR time and increased accuracy-not backed up by numbers
Sigron et al. 2020 [30]	Models (Orbit for pre-contoured Ti mesh)	In-house	Surgeon (Surgical resident)	Mimics Innovation Suite v. 20-21-L	P: MakerBot Replicator+- FDM Stratasys Objet30 Prime- PolyJet	Total time (segmenting and preparing data for 3D - printing): < 1 h	NA (No printing time was clearly mentioned)	OR time-> Intervention Group: 57.3 +/- 23.4 vs Conventional Group 99.8 +/- 28.9;<br>Using pre-bent implants can reduce OR time by 42.5 min	Material cost/mode: PLA-2$, Med610-25$; Surgical theater cost/min is 47.5-103$ and method reduces OR time by 42.5 min=> economy of 4377.50$	Pre-bent Ti meshes are more accurate than freehand bent Ti meshes. Significant reduction of surgery time, thus cost efficient.<br>(All backed up by numbers and statistics)
Narita et al. 2020 [31]	Models (Orthognathic Surgery)	In-house	Surgeon (suggested)	Volume Extractor 3.0, POLYGONALmeister Ver.4-L	P: Value3D MagiX MF-2000-FDM	Panning and printing time not separated	Mean fabrication time: 12 h 14m	OR time: With 3D models-226 ± 18 min vs. No	Average weight of model is 165g- >5$/model;	Reduced OR time; Reduced bleeding (252.2 ± 97.7 g- With 3D

				(undisclosed/ unavailable data on license)			(planning and printing)	3D models-260 ± 36 min	Not included: labor, printer, software costs.	models vs 331.2 ± 85.9 No 3D models
Lassausaie et al. 2020 [32]	Zygomatico-maxillary model (For surgery planning and plate bending)	In-house ("office-based 3D printing"	Surgeon (suggested)	3D Slicer, Meshmixer-OS	Zortrax M200-FDM	NA	NA	Pre-bended plates facilitate the osteosynthesis step reducing OR time- not backed up by numbers	NA	PO orbital volume was significatively reduced as planned (slight difference persisted between both sides - Right orbital volume PO: 24.21 cm3, Left orbital volume: 22.12 cm3
Menzel et al., 2020 [65]	Orbital floor mold ("form-box") and anatomical models	In-house	Surgeon (suggested)	Mimics inPrint ; 3-matic-L	P: Form 2,-SLA; M: Dental SG V1 resin	Planning: from importing data to the ready-to-print file: 2 h;(out of which 45 min. to design the "form-box")	Printing time: 4 objects (fractured orbit, mirrored orbit, upper and lower part of the ''form-box''): 11.5 h	NA	100 E/case for processing software license rights and printing material; Polydioxanone foil- 250 E	Patient specific polydioxanone foil showed a very good passive fit under optical and haptic control by 2 oral and maxillofacial surgeons
Williams et al. 2020 [68]	"in-house" printed dental prosthesis to be used with outsourced gear for mandible reconstruction.	In-house: prosthesis Outsourced: rest of the gear for mandible reconstruction.	Biomedical engineer for the VSP; followed by surgeon for the prosthesis	Blue Sky Plan (free license, pay per STL export); Meshmixer-L	Form2, Phrozen Shuffle-SLA	After digital fibula construct was provided, in 24h the prosthesis was designed and printed	Prin time: Prostheses will require 1 to 2 h	NA	P: 500-10.000$; Blue Sky Plan- 25$/ STL export; M: 8.34$ of resin/prosthesi s; Initial cost of printer and supplies can be <3000$	All implants were in restorable positions, although 3 were not included in the prosthesis for 3 patients. Implant integration rate of 93% at 3-6 months.
Tel et al. 2020 [60]	3d printed mold for cranioplasty plate	In-house- (university center-Virtual Surgical Planning and 3D Printing Lab)	Surgeon (Surgical team)	Mimics ;3-Matic-L	Form 2-SLA	Mold production, from DICOM to final printing: 48 h	Mold production: 48 h; Production time (CT to surgery) dropped from 20 or 30 d to only 2 D	Mean OR time was 137.2 min.	Software cost: 21,000$/y (segmentation, design, analysis modules) M: Grey Pro resin- $192/ 1L	3D analyses validate accuracy of reconstructions - based on numbers and statistics

Gomez-Feria et al. 2021 [38]	Head models	In-house	NA	MRIcron, MRIcroS, Meshmixer-OS	P: BQ Witbox 2-FDM; M: PLA, flexible polyurethane	NA	3D printing process took 48 h	NA	Material cost: 30$	Survey from 8 surgeons strongly supports the use of this model for surgical planning, IO surgical guidance, patient communication, surgical training.
Beckers et al. 2021[50]	Implant positioning guide for ear epenthesis	In-house	NA	ProPlan CMF, Geomagic Sculpt-L	P: MakerBot Replicator-FDM	NA	NA	NA	NA	NA
Ostas et al.2021[51]	Cutting guide; Bone fragments positioning guide	In-house	Surgeon	MIMICS, 3-matic Research-L	P: Zortrax M300 Dua-FDM; Form 2-SLA; M: PLA Surgical Guide Resin V1	Model and cutting guides design: 3.5 h; bone fragments positioning guide design: 1.2 H	Prin time: SLA - 3.8 h (2.5 h printing, 1.3 h for postprocessing). FDM-mandibular model for pre-bending was printed in 10.9 h (1 h for post-processing)	NA	P: FDM- 4309 EUR; SLA- 5580 M: PLA-42 E/800g, Soluble filament-140 E/ 800g; Form 2 resin 280E/L	Experimental surgery model was compared with the virtual planned reconstruction-> accuracy of the hospital-based workflow is similar to previously published studies - based on numbers
Tel et al. 2021 [61]	Modular mold system to perform craniofacial reconstruction	In-house	Surgeon	Mimics Innovation Suite version 23.0-L	P: Form 2-SLA	NA	NA	OR time was given (table 1 in paper) for every surgery without any indication of OR time reduction.	NA	The IO prosthesis production was successful, it did not prolong OR time; No implant-related infections; No temporal hollowing cases; No abnormal depression or bulging along orbital rim. - not baked-up by numbers
Mascarenhas et al. 2021[52]	Positioning guide (Orthognathic splint for singlejaw cases)	In-house	Surgeon	Dolphin Imaging software-L	P: Form 2-SLA	Average time for splint design: 4.5 min.	Total time (initial scan to fabrication)/ case: 5-9 h (time for RP depends on complexity/ number of prints)	NA	Cost per orthognathic Splint: 0.73$ CAD (The cost of the resin used to print each splint)	Increased intraoperative efficiency, and reliable and accurate outcomes - just as a citation, not backed-up numbers or statistics

Schön et al. 2021 [69]	Cranioplasty template models	In-house	NA	Mimics, 3-matic-L	Stratasys Objet 30 Prime-PolyJet	NA	Printing time for larger implants is around 5 h (based on previous study)	Median (range) OR time was 121 (89-206) min.	Overall cost of implant Manufacturing : 250$/ implant (excluding working 1-2 hours, costs for hardware, software, licensing	Superimposing the reconstructed CT data or by mirroring of the non-affected side with the CT data after cranioplasty demonstrated excellent congruency with the patients’ tabula externa.
Akiki et al. 2021[33]	Model (Orbital model for IO molding of resorbable implants)	In-house	NA	3D Slicer, Blender-L	P: Stratasys J750-PolyJet	NA	NA	NA	NA	PO patient had intact vision and discharged without complications; Resolution of diplopia within two weeks; No evidence of enophthalmos; no evidence of vertical asymmetry or hypoglobus.
Maglitto et al. 2021 [53]	(1) Fibula and mandibulae cutting guide; (2) orbital mesh conformer; (3) zygomatic shield; (4) covid-19 mask and face shield	In-house	Surgeon (suggested)	Invesalius, Meshmixer-OS	P: Ultimaker 2 and Extended+-FDM; M: TPU, PLA	(1)-design was about 3 h	(1)- printing process and sterilization 6 h; (4)- to print the mask body- 6 h, filter slot- 1 h and face shield-3 h.	NA	(1)- 6 m of PLA filament-3.6 E/case (4) - final device (covid-19 mask) < 5 E	(1) 3D volume overlap-> standard deviation 5.496 mm (1.966 - 8.024 mm) (2) good overlap between the PO planning and the postop. result-backed-up by numbers and statistics (3) postop. CTs revealed excellent positioning of the device and a correct fracture reduction; (4) mask was considered to be highly reliable (based on survey)
Dvoracek et al. 2021 [34]	Models (orbit)	In-house (Children’s Hospital of Pittsburgh Department of Radiology 3D	NA	Mimics, 3-Matic-L	P: Form 2-SLA; M: Dental SG resin	Average model preparation: 10.4 h	Average model preparation: 10.4 h	Average case duration 2.0 h	Material cost/ patient: $21	Excellent implant conformation on postop. CT; 3 postop. complications (not implant-related). PO difference in bilateral orbital volumes

		Printing Laboratory)								was significantly greater than postop. difference ($p < 0.01$)
Cole et al. 2021 [70]	3D printed calvarium-anchored ventricular catheter occlusion device	In-house (Suggested title:" Local 3-Dimensional Printing of..."	Surgeon and engineer	SolidWorks-L	P: Form 2-SLA M: Durable V2 resin	NA	NA	NA	NA	Device eliminates the need for a surgical assistant to occlude and retain the intracranial catheter.
Sharma et al. 2021 [71]	Cranial implants	In-house	NA	MIMICS, 3-matic- L	P: Apium M220-FFF M: PEEK	NA	NA	NA	NA	Implants: -had high dimensional accuracy; revealed clinically acceptable fit; had strength for clinical use; variability between peak load was noticed- backed-up by numbers and statistics
Šimić et al. 2021 [54]	Cutting guides (fibula and mandibular)	In-house	Surgeon	3D Slicer, Blender-OS	P: Stratasys Objet30-PolyJet M: Med620	Cutting-guide modelling: 15 min or less (others report 50 min or higher)	NA	NA	NA	Without cutting guides, work would be less precise and time consuming-mentioned in discussions, not backed-up by numbers and statistics
Sigron et al. 2021 [35]	Models (orbit)	In-house	NA	Mimics Innovation Suite-L	P: MakerBot Replicator+-FDM; Objet30 Prime-PolyJet M: PLA, Med610	NA (Included in the entire production time)	Virtual planning, 3D-printing, and PO mesh modelling: < 2 h	Surgery reduced by 35.9 min. (intervention group) - backed up by numbers and statistics	NA (Evaluation of the cost savings has not been feasible due to different standards)	Prebent meshes reduce OR time, do not delay surgery, have slightly more effective ophthalmic outcomes-backed up by numbers and statistics
Postl et al. 2021 [55]	Surgical guides (For biopsies of the lower jaw)	In-house	NA	Mimics Innovation Suite-L	P: ZPrinter 650 –Inkjet (variant of CJP); Form 2-SLA	NA (Planning and printing time taken together)	Surgical guide fabrication: 128 ± 17 min.	NA (Not clinical)	NA	Surgical guides allow significantly higher accuracy of biopsies-backed up by numbers and statistics

Bergeron et al. 2021 [36]	Models (cranial vault, mandible, orbit)	In-house (in-house printing, planning both in-house and outsourced)	Surgeon (surgeons / surgeon + technician when outsourced)	MeshMixer-OS	P: Ultimaker, Materio3D-FDM	NA	Overall in-house printing phase/model ranged from 2 h 36 min. - 26 h 54 min. (mean = 7h 55 min)	NA	Printer-3500$; Filament cost/model: $0.20 and $2.65USD (mean = $0.95)	Models were accurate; prebent plates were used without modification once placed; There were no operative takebacks due to implant shape. - no numbers and statistics
Ritschl et al. 2021 [56]	Cutting guides/ repositioning aids (For fibula ad mandible)	In-house	Surgeon	Mimics-L, Blender-OS	P: Form 2-SLA M: resin (Dental SG)	Planning time: 5-6 h	Printing: 180 min. (120–255)/case; Total time (plan, print, postprocessing, drill sleeves preparation, sterilization): 2-3 d	The median overall OR time: 650 min. (480–840), ischemic time 165 min. (90–240)	Median costs / case were 14.30 E (4.50–25.30).	Open-source software facilitates cost-effective, precise in-house virtual planning of mandibular reconstructions- backed up by numbers and statistics- see full text for further data
Pöppe et al. 2022 [62]	Mold (For cranial plate)	In-house	Surgeon	Brainlab iPlan, Mimics inPrint-L	P: Form 3B or Form 2-SLA M: resins (Dental SG Resin, Surgical Guide Resin).	Preparation time for a cranial implant: 30 min.	Printing: template ring (3-5 h); template mold (5-8 h) Postprocessing: 15 min.	OR time: mean overall skin-to-skin time: 92 min.	Expenses/ implant: 300 E Commercial option: 5.000-10.000 E => 95% cost reduction/implant	Reduced OR time, costs (calculated and compared with data from literature); Patients/legal representatives satisfied with skull shape and cosmetic appearance"

OS-open source, NA-not available, h-hours, OR-operating room, cc-cubic centimeters, $-American dollars, AVM-arterio-venous malformations, L-licensed, FFF-fused filament fabrication, IT-information technology, FDM-fused deposition modeling, min.-minutes, PLA-polylactic acid plastic filament, PVA-Polyvinyl Alcohol, M-material, P-printer, ABS-Acrylonitrile butadiene styrene, g-grams, IO-intraoperative, PO-postoperative, 3D- three-dimensional, SLS- selective laser sintering, E- euro, SG-surgical guide, y-years, CT-computer tomography, PMMA- polymethyl methacrylate, PSI-patient specific implant, PEEK – Polyetheretherketone, MJP- MultiJet Printing, S-software, BJ-Binder Jetting, DICOM-Digital Imaging and Communications in Medicine, STL- Standard Tessellation Language, SLA-stereolithography , d-days, DLP- Digital Light Processing, vs.-versus, ORIF-open reduction and internal fixation, £-sterling pound, SLM-selective laser melting, VSP-virtual surgical planning, CAD-Canadian dollars, CAD-CAM- computer assisted design - computer assisted manufacturing, VP-Virtual planning, RP- rapid prototyping, TPU- thermoplastic polyurethane, CJP- Color Jet Printing (CJP).
